# Nebulisation of Receptor-Targeted Nanocomplexes for Gene Delivery to the Airway Epithelium

**DOI:** 10.1371/journal.pone.0026768

**Published:** 2011-10-26

**Authors:** Maria D. I. Manunta, Robin J. McAnulty, Aristides D. Tagalakis, Stephen E. Bottoms, Frederick Campbell, Helen C. Hailes, Alethea B. Tabor, Geoffrey J. Laurent, Christopher O'Callaghan, Stephen L. Hart

**Affiliations:** 1 Wolfson Centre for Gene Therapy of Childhood Disease, UCL Institute of Child Health, University College London, London, United Kingdom; 2 Centre for Respiratory Research, University College London, London, United Kingdom; 3 Department of Chemistry, University College London, London, United Kingdom; 4 Department of Infection, Immunity and Inflammation, University of Leicester, Leicester Royal Infirmary, Leicester, United Kingdom; University of Helsinki, Finland

## Abstract

**Background:**

Gene therapy mediated by synthetic vectors may provide opportunities for new treatments for cystic fibrosis (CF) via aerosolisation. Vectors for CF must transfect the airway epithelium efficiently and not cause inflammation so they are suitable for repeated dosing. The inhaled aerosol should be deposited in the airways since the cystic fibrosis transmembrane conductance regulator gene (*CFTR*) is expressed predominantly in the epithelium of the submucosal glands and in the surface airway epithelium. The aim of this project was to develop an optimised aerosol delivery approach applicable to treatment of CF lung disease by gene therapy.

**Methodology:**

The vector suspension investigated in this study comprises receptor-targeting peptides, cationic liposomes and plasmid DNA that self-assemble by electrostatic interactions to form a receptor-targeted nanocomplex (RTN) of approximately 150 nm with a cationic surface charge of +50 mV. The aerodynamic properties of aerosolised nanocomplexes produced with three different nebulisers were compared by determining aerosol deposition in the different stages of a Next Generation Pharmaceutical Impactor (NGI). We also investigated the yield of intact plasmid DNA by agarose gel electrophoresis and densitometry, and transfection efficacies *in vitro* and *in vivo*.

**Results:**

RTNs nebulised with the AeroEclipse II BAN were the most effective, compared to other nebulisers tested, for gene delivery both *in vitro* and *in vivo*. The biophysical properties of the nanocomplexes were unchanged after nebulisation while the deposition of RTNs suggested a range of aerosol aerodynamic sizes between 5.5 µm–1.4 µm cut off (NGI stages 3–6) compatible with deposition in the central and lower airways.

**Conclusions:**

RTNs showed their ability at delivering genes via nebulisation, thus suggesting their potential applications for therapeutic interventions of cystic fibrosis and other respiratory disorders.

## Introduction

Cystic fibrosis (CF) is one of the most common autosomal recessively inherited disorders with a carrier frequency of 5% in the Caucasian population [1], [2]. CF predominantly affects the exocrine epithelium in a number of secretory tissues and organs. Respiratory insufficiency is the major cause of CF mortality. The defect is caused by mutations in the gene for the cystic fibrosis transmembrane conductance regulator (*CFTR*) [3], [4], which is a cAMP-activated chloride channel in the apical membrane of the surface epithelium (for review see [5]). The mutated *CFTR* causes an ion imbalance leading to a reduced volume of the airway surface liquid, mucus dehydration and reduced mucociliary clearance [6]. This provides a favourable environment for bacterial infections leading to a progressive decline of lung function [7].

The aim of CF gene therapy is to deliver the corrective gene to the airway epithelium to restore chloride channel activity. However, several gene therapy trials with vectors including adenovirus (Ad), adeno-associated virus (AAV) and liposomes did not achieve clinically relevant levels of *CFTR* gene transfer [7], [8]. In addition, the administration of adenoviral vectors [9], [10] and AAV [11], [12] augmented inflammation, due to the presence of neutralizing antibodies and T cell responses in the already-compromised respiratory system. The aerosol administration to the lungs of a cationic liposome, GL67, complexed with plasmid DNA caused febrile flu-like symptoms in some patients [13], [14].

We have shown previously that a novel nanocomplex formulation was effective at delivering genes to the lungs of mice by direct instillation [15]. These receptor-targeted nanocomplexes (RTNs) comprised peptides (K_16_GACSERSMNFCG), cationic liposomes (DHDTMA/DOPE) and plasmid DNA. The peptide motif SERSMNF displays close similarity to receptor binding proteins of two intracellular pathogens, rhinovirus and *Listeria monocytogenes* [16]. Rhinoviruses of the major group bind intercellular adhesion molecule-1 (ICAM-1, CD54) [17], which is upregulated in chronically inflamed airway epithelium [18], [19], hence suggesting it could be an appropriate receptor target for CF vectors.

Nebulisers are used extensively for the administration of medication by inhalation, typically generating aerosol particles less than 5 µm in diameter that can reach the lower respiratory tract. Thus nebulisation is an attractive option to deliver genes directly to the affected epithelial cells in the lungs of patients with CF. A crucial step towards this goal is to find an appropriate formulation and a compatible nebuliser. Vector suspensions should maintain their stability and biophysical characteristics, protecting the plasmid DNA from shearing forces during nebulisation so that its biological activity is preserved. In addition, the yield from the nebuliser must be maximised [20]. A careful evaluation of the delivery device is therefore an essential requirement for designing a CF gene therapy protocol. Different types of nebuliser technologies are available commercially including jet, vibrating mesh and ultrasonic nebulisers. In the present study we assessed the delivery of RTN suspensions with jet and vibrating mesh nebulisers, as the ultrasonic devices are not suitable for liposome-based vectors [21].

The aerodynamic particle sizes of RTN aerosols were determined by sample deposition in a Next Generation Pharmaceutical Impactor (NGI). The stage at which the aerosol is deposited in the seven-stage cascade impactor, reveals the terminal settling velocity of the nebulised suspension dependent upon the aerodynamic diameter (specific density, shape and gravity) of the aerosolised particles [22].

RTN formulations in an AeroEclipse II BAN nebuliser displayed particle stability following nebulisation and retained transfection efficiency *in vitro* and *in vivo*, while aerosol aerodynamic sizes were compatible with deposition in the airways and in the lung. Therefore, RTN formulations delivered by an AeroEclipse nebuliser showed the characteristics desirable for CF gene therapy.

## Results

### Influence of the nebuliser on RTN delivery

Although convenient for administration of therapeutics to the airways, the nebulisation process of transforming a liquid medication into a respirable mist can involve strong shearing forces, particularly in jet nebulisers. These devices are usually operated until no more useful aerosol is generated, although in jet nebulisers a residual volume of the suspension usually remains in the sample reservoir. We examined the aerosolisation of the RTN suspensions, based on the LED-1 formulation described previously [15] comprising a cationic liposome (DHDTMA/DOPE), a peptide E (K_16_GACSERSMNFCG) and a plasmid DNA using different nebulisation systems and focused on their suitability for treating CF patients. At the transfection weight ratio of 1.5∶4∶1 (L∶P∶D), the formulation contained, per plasmid, approximately 2,750 peptide molecules, 4,550 molecules of DHDTMA and 3,750 molecules of DOPE. The calculated N/P ratio was approximately 5, supporting the observation that the nanocomplexes were positively charged.

Two jet nebulisers, the AeroEclipse II BAN and PARI-LC Plus, which use rapidly expanding compressed air to atomise drug solutions and suspensions, were compared with a vibrating mesh nebuliser, the Aeroneb® Pro. The vibrating mesh nebuliser has the potential advantage of lower shearing forces and a more efficient aerosolisation process, with a negligible residual volume following nebulisation. In comparison experiments, all the nebulisers were operated for 10 min and the AeroEclipse was used in open vent mode which allows for prolonged admission of gas through the vent which results in shrinkage of the aerosol droplets due to evaporation, increasing the nebuliser performance [20]. The aerosolised nanocomplex suspension was collected from the NGI. The air flow rate in the NGI was 15 L/min and the equipment was chilled before each experiment to reduce evaporation of the deposited material during the experimental procedure [23].

**Figure 1:**
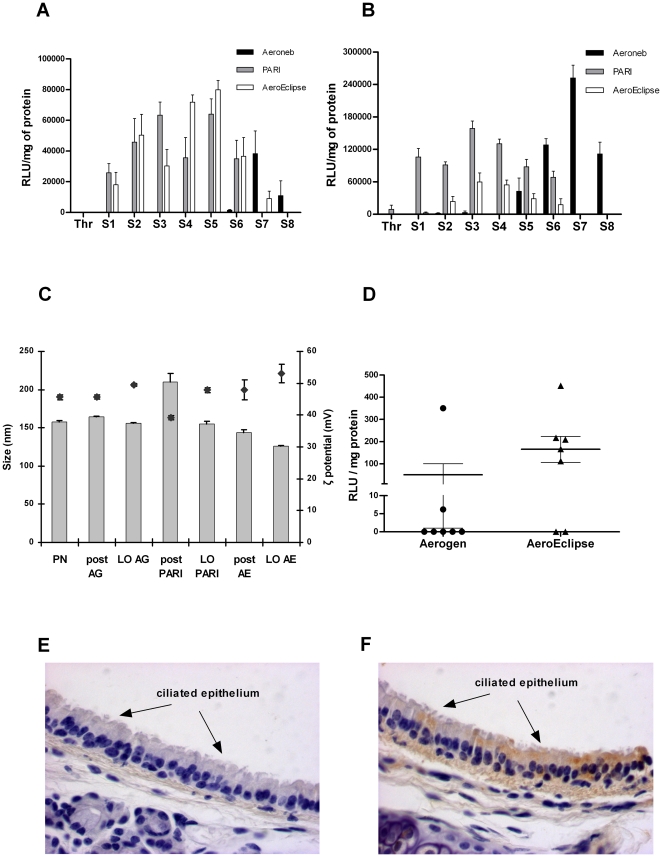
Comparison of vector delivery with three nebulisers (AeroEclipse II BAN, PARI-LC Plus and Aeroneb® Pro). **A and B Transfections of 16HBE14o- and CFBE41o- cells mediated by RTNs nebulised through the NGI:** RTNs were made in clinical grade H_2_O at weight ratio of 1.5∶4∶1 of DHDTMA/DOPE (1∶1 molar ratio), peptide E and pCpG-free *lacZ* plasmid DNA, respectively. 3 ml of RTN suspension was used for each nebulisation and all three nebulisers were operated at a flow rate of 15 L/min for 10 min. After aerosolisation nebulised nanocomplexes, deposited in the different stages of the NGI, were collected by adding to each stage 1 ml of water. A volume of the collected samples, diluted in OptiMEM, was added onto cells. The expression of the reporter gene was assessed 48 h after delivery. Representative results of transfection experiments in both cell lines for each nebuliser (AeroEclipse II BAN, PARI-LC Plus and Aeroneb® Pro) are shown. The error bars represent the standard error of the mean of quadruplicate wells. Nebulisations through the NGI were repeated 3 times with each nebuliser for both cell lines and cumulative results are reported in Table 1. RLU = Relative Light Unit; Thr = throat; S1–S8 = stages 1–8 of the NGI, S8 = micro-orifice collector. **C Physicochemical properties pre- and post-nebulisation:** The size and the ζ (electrokinetic or zeta) potential were measured using a Malvern Nano ZS Zetasizer on aliquots of pre-nebulised nanocomplexes, of nebulised samples collected in a 50 ml tube, and of residual RTN suspension remaining in the nebuliser chamber upon completion of the nebulisation. The average values of three independent measurements of each sample were automatically performed. Bars represent the average values of the sizes of two independent experiments ± the standard deviation whereas the diamonds indicate the ζ potentials in the same samples ± the standard deviation. PN = pre-nebulisation RTN suspension; post = post-nebulised nanocomplex suspension; LO = (leftover) RTNs retained in the nebuliser chamber. **D** ***In vivo*** **delivery of RTNs containing luciferase reporter gene in C57BL6 mice:** 2 ml of RTNs, prepared with pCILuc plasmid, were nebulised using either the Aeroneb® Pro or the AeroEclipse II BAN, into a Plexiglas chamber in which 9 mice (C57BL6 strain) were confined. 24 h later, 7 of the murine lungs were then analysed for luciferase expression as described in the methods. Luciferase activity is expressed as Relative light Unit (RLU) per milligram of protein. There was not statistical difference between the two groups when the readings were normalised to protein content, but there was a statistical difference (p<0.05) in the luciferase activity (Mann-Whitney test). **E and F Localisation of luciferase expression by immunohistochemistry:** Trachea of C57BL6, following a single nebulisation of 2 ml of RTNs containing pCILuc were stained with 3,3′-diaminobenzadine (F), and counterstained with haematoxylin. The transfected tissue was localised in the ciliated airway epithelium whereas the negative control section (E) was not probed with the primary antibody. Objective magnification: 40×.

The average output efficiency of RTNs from the three different nebulisers, compared by quantifying DNA in the nebuliser chamber prior and after completion of the aerosolisation, was approximately for the Aeroneb 43%, PARI 86% and AeroEclipse 85%, respectively (n = 2). The transfecting activity of the aerosolised nanocomplexes, collected from the different stages of the NGI, was measured in normal bronchial epithelial (16HBE14o-) cells (Figure 1A and Table 1) or CF bronchial epithelial (CFBE41o-) cells (Figure 1B and Table 1), after diluting them with OptiMEM serum-free medium. The β-galactosidase activity measured in cell lysates transfected with samples from the different stages of the NGI suggested that the Aeroneb generated smaller aerosol droplets than the other two nebulisers, with most activity recovered from stage 7 (particles with an aerodynamic diameter of less than 0.98 µm) and the micro-orifice collector (S8), while in CFBE14o- cells expression was also found in stages 6–8, with peak activity in stage 7 (Figure 1A, 1B and Table 1). The pattern of nanocomplex distribution was comparable for both the AeroEclipse and PARI jet nebulisers, where the vast majority of the transfection activity was found in the aerosol particles deposited between stages 2 to 6 representing aerodynamic diameters of 8.6 µm to 1.4 µm, respectively.

**Table 1:** Transfection values (RLU/mg of protein) of RTNs nebulised through the NGI with either Aeroneb® Pro, PARI-LC Plus or AeroEclipse II BAN.

NGI stage:		Thr	S1	S2	S3	S4	S5	S6	S7	S8
NGI cut-off (µm):			*14.1*	*8.61*	*5.39*	*3.30*	*2.08*	*1.36*	*0.98*	
**Aerogen**	**16HBE14o- cells**	8819±8512	44057±42078	48435±48435	52800±52800	54990±54409	82708±61014	85688±47966	81088±62850	27785±22544
	**CFBE41o- cells**	2283±2119	17841±17841	16493±15717	20369±18925	40183±40183	70722±50388	104611±49689	111347±74199	39720±35960
**PARI-LC**	**16HBE14o- cells**	9702±6320	109584±46294	170307±71301	154341±46768	124654±44125	150031±44334	133399±53240	57±57	0±0
	**CFBE41o- cells**	8021±4254	123006±75374	118428±72533	106097±43713	103535±43981	86546±37031	81606±23286	95±40	9±6
**AeroEclipse**	**16HBE14o- cells**	61584±61444	89623±72874	110145±50372	110436±47498	96887±24154	116254±47744	137416±52193	3755±3117	38±38
	**CFBE41o- cells**	49870±49574	149647±139462	68054±53329	81675±44578	82028±37761	69090±31892	76312±55886	711±558	16±10

Thr = throat; S1–S8 = stages 1–8 of the NGI, S8 = micro-orifice collector. All three nebulisers connected to the NGI were operated for 10 min at an air flow rate of 15 L/min as described in methods. The transfection results are average values of three independent experiments, each one was carried out in quadruplicate, and the error bars represent the standard error of the mean.

Samples from the Aeroneb showed no difference in either the sizes (160 nm) or the ζ (electrokinetic or zeta) potentials (+46 mV) between the post-nebulisation residual material in the nebuliser reservoir (LO) and the suspension prior to nebulisation (PN) (Figure 1C). However, the RTNs nebulised with the PARI-LC Plus were larger post-nebulisation (210 nm) than pre-nebulisation (158 nm) with the ζ potential decreased from +46 mV to +39 mV (Figure 1C). RTNs collected from the AeroEclipse post-aerosol and the leftover in the nebuliser chamber (Figure 1C, last two bars and diamonds), showed a slight decrease in the geometric sizes (144 nm and 126 nm, respectively) and a slight increase in the ζ potential (+48 mV and +53 mV, respectively).

To further compare the Aeroneb® Pro and the AeroEclipse II BAN, an *in vivo* study, with nine mice per cohort, was performed by whole-body nebulisation with a 2 ml single dose of RTNs containing the luciferase reporter gene plasmid pCILuc at a concentration of 160 µg/ml of pDNA. Luciferase assays of lung extracts (n = 7/group) at 24 h after aerosolisation indicated that the AeroEclipse was the most efficient nebuliser for *in vivo* delivery to mice (Figure 1D). Indeed, 71% of the C57BL6 mice (5/7) showed luciferase expression in their lungs, versus 29% (2/7) of those aerosolised with the Aeroneb (one of which was positive at very low level). Values of luciferase activity (Relative Light Unit) from the lungs of mice nebulised with the AeroEclipse were statistically different at 0.05 level, compared to those aerosolised with the Aeroneb, however when normalised to the protein concentration there was no difference between the two groups. Lungs from the two remaining mice were used for immunohistochemical localisation of luciferase. Luciferase enzyme was detected predominantly in ciliated tracheal epithelial cells following nebulisation with the AeroEclipse (Figure 1F). No positive staining was observed in the lower airways or parenchyma of either mouse. No staining was observed in sections from naïve mice or in sections of lung from nebulised mice where incubation with the primary antibody was omitted (Figure 1E).

In summary, the RTN formulations nebulised with the AeroEclipse II BAN, preserved almost unchanged the parameters of the colloidal suspension and was the most effective nebuliser *in vivo* and therefore was used for further investigations. The Aeroneb® Pro, distributed to later stages of the NGI (particles with an aerodynamic diameter less than 2 µm) and presumably would deposit aerosol deeper in the lung *in vivo.* It also tended to block during nebulisation making its use problematic. The nanocomplexes nebulised through this device remained intact by particle size measurements thus indicating a preserved activity, but its efficacy *in vivo* was not as good as the AeroEclipse. The PARI-LC Plus showed some alterations in the physical properties of the suspension nebulised and so was not investigated further.

### Yield of DNA from nebulised nanocomplexes

**Figure 2:**
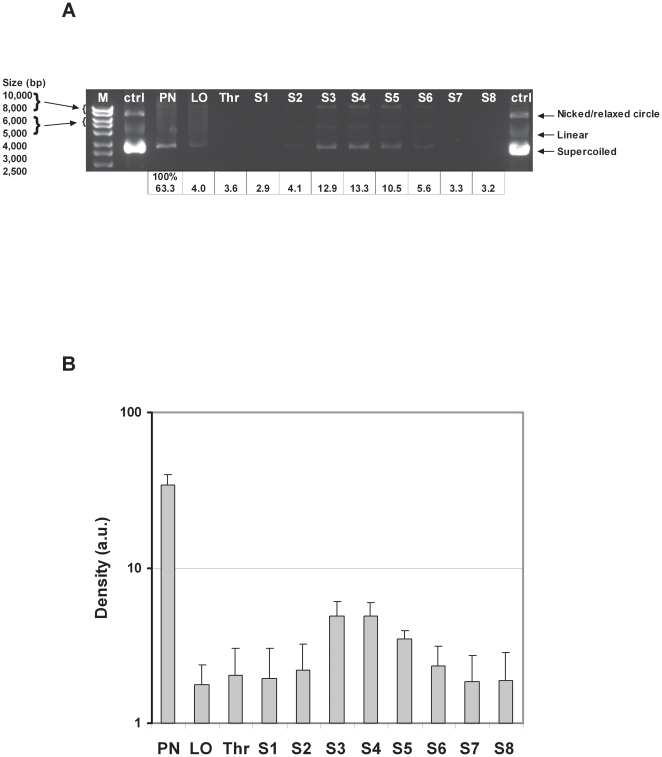
Analysis of the DNA recovered from nanocomplexes collected from the NGI stages. **A Visualisation of bands on agarose gel:** After nebulisation of RTNs through the NGI with the AeroEclipse, the DNA (pEGFP-N1 plasmid) was released from samples collected from each stage, the throat (Thr), as well as the suspension pre-nebulisation (PN) and the leftover (LO) in the nebuliser reservoir. The released DNA was electrophoresed on agarose gel, alongside the original plasmid used to form the complexes (ctrl), and visualised by EtBr staining under UV light. A representative experiment is shown. The total yield of DNA of the experiment shown was 63% and the yield relative to each band of DNA in the gel is also indicated underneath. **B Quantification of bands of DNA released from the RTNs:** Nanocomplexes prepared at the DNA concentration of 160 µg/ml were nebulised through the NGI and the RTNs pre-nebulisation (PN), the leftover in the nebulisation chamber (LO) or deposited in the throat (Thr) or in the various stages (S1–S8) were collected. The DNA extracted from the samples was analysed by agarose gel electrophoresis. The agarose gel images acquired were analysed by ImageJ. To compare the density values per area, the region of interest (ROI) of each independent experiment was normalised to 1, before calculating the mean values. The experiments were performed twice and the error bars represent the standard error of the mean.

To better characterise the RTN suspension nebulised by the AeroEclipse, DNA degradation and/or particle disruption was then assessed. RTN suspension (3 ml) was nebulised into the NGI and samples were collected from the different stages by rinsing each cup and the throat with 1 ml of water. DNA was released from the nebulised nanocomplexes and non-nebulised suspensions by detergent treatment, then analysed by agarose gel electrophoresis and the DNA was quantified by densitometry. The amount of DNA recovered was higher from stages 3–5 (aerodynamic diameter of 5.4 – 2.1 µm, respectively), though stage 6 (1.4 µm) showed some positivity (Figure 2A). The packaging of DNA in the suspension of liposomes and peptides appeared to protect the DNA from breakage. In a few experiments we noticed a slight increase in the amount of nicked/relaxed circles, but this could have arisen during the sample processing.

Quantification of the plasmid DNA bands by densitometry, expressed as a percentage of the total (PN), indicated that the yield of nebulised nanocomplexes obtained from the NGI was 63% of DNA (Figure 2A). The vast majority of the DNA was found in stages 2–6 (8.6 µm – 1.4 µm), accounting for about 45% of the total DNA nebulised and approximately 70% of that recovered after nebulisation.

The distribution of quantified DNA from two independent experiments (Figure 2B) followed essentially the same pattern of a single experiment shown in the gel in figure 2A. The yield of DNA collected from the NGI compared to the starting material on average was around 77%, for the nanocomplexes, at a DNA concentration of 160 µg/ml, formulated in H_2_O.

### The physical size and stability of the nebulised nanocomplexes collected from the NGI

**Figure 3:**
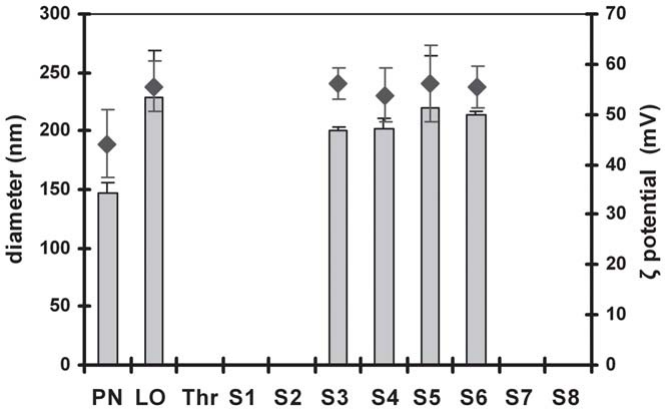
Geometric diameters and electrokinetic potentials (ζ) of RTNs before and after nebulisation through the NGI. The aliquots of the same samples nebulised through the NGI (as in Figure 2A) were used to measure the size (bars) and the ζ potential (diamonds) using the Malvern Nano ZS Zetasizer. The average values of three independent measurements per each sample were automatically performed. Results of two independent experiments ± the standard deviation, from samples providing accurate readings are shown: PN = pre-nebulisation RTN suspension; LO = (leftover) RTNs retained in the nebuliser chamber upon completion of the nebulisation and samples from stages 3–6 of the NGI.

The average geometric size of the nanocomplexes from the aerosol collected in the NGI cups was compared to pre-nebulised samples to further determine the stability of the nanocomplexes to nebulisation. In this experiment the nanocomplexes increased in size after nebulisation, from 150 nm to about 200–230 nm (Figure 3), suggesting that the increase of the size may have occurred during the passage of the aerosolised suspension through the NGI. The ζ potential also increased from +45 mV to +54–56 mV indicating that the nanocomplexes bearing a higher colloidal stability were preferentially aerosolised.

### Quantification of the cells transfected

**Figure 4:**
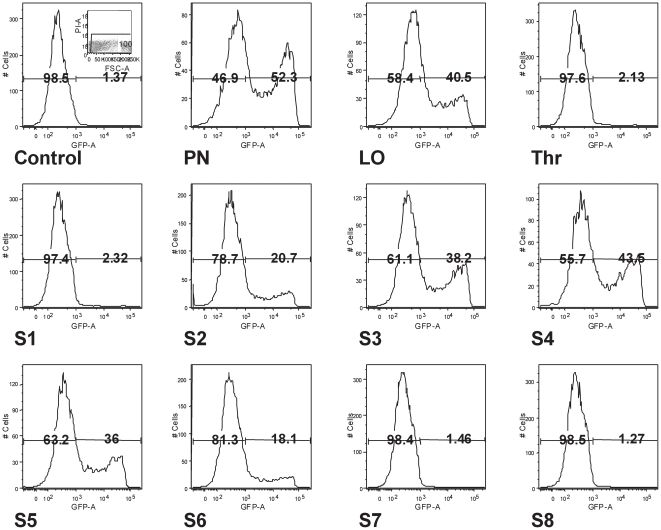
Transfection efficiencies mediated by RTNs carrying pEGFP plasmid and assessed by flow cytometry. RTNs were formed using pEGFP-N1 plasmid DNA. Aliquots of nanocomplexes nebulised through the NGI (as in Figure 2A and 3) were used to perform transfections. After 48 h incubation, cells were harvested and analysed by flow cytometry to determine the level of expression of enhanced green fluorescent protein (EGFP) in the samples collected from each NGI stage. A representative experiment carried out in normal human bronchial epithelial cell-line 16HBE14o- is shown (cumulative experiments are summarised in Table 2). The dot-plot profile of untransfected cells (ctrl) represents the population selected as alive whereas the 1st panel of histogram profile shows where the markers to discriminate the EGFP positive from negative population were set. On each panel the percentage of EGFP negative (left) and EGFP positive (right) cells is indicated. PN = represent cells transfected with pre-nebulisation RTN suspension; LO = cells treated with leftover nanocomplexes in the nebuliser chamber after nebulisation, Thr = throat, and S1–S8 cells transfected with samples from the different NGI stages, respectively.

The samples retrieved from the different stages of the NGI were also used to determine their post-nebulisation ability to transfect cells, using the enhanced green fluorescent protein (EGFP) reporter gene. Equal volumes of nebulised material were added to cultures of normal (16HBE14o-) or CF (CFBE41o-) bronchial epithelial cells to detect the EGFP expression by flow cytometry. The viable cells were selected by staining with propidium iodide as shown in the control panel. The percentages of live EGFP-transfected cells at each stage of the NGI are shown in comparison with the EGFP-negative population. Pre-nebulisation suspension (PN) and nanocomplexes remaining in the nebulisation chamber upon completion of the nebulisation (LO) transfected the highest number of cells, while fewer positive cells were found in transfections performed with material collected from the throat and stages 1, 7 and 8. The number of cells transfected seemed to display a normal distribution trend from stage 2 (aerodynamic diameter of 8.6 µm) to stage 6 (1.4 µm), having its maximum peak at stage 4 (aerodynamic diameter cut-off of 3.3 µm). Flow cytometry profiles of 16HBE14o- cells transfected with pEGFP (Figure 4) clearly demonstrated that the distribution of EGFP-positive cells transfected with nanocomplexes recovered from the NGI, correlated with the transfection data (Figures 1A and B) and the amount of DNA recovered (Figure 2). Similar results were obtained for the same experiment repeated in 16HBE14o- cells and in two independent experiments in CFBE41o- cells (Table 2).

**Table 2:** Percentage of cells transfected by samples collected from the NGI and assessed by flow cytometry.

Percentage (%) of transfected cells ±S.E.M. :	Ctrl	PN	LO	Thr	S1	S2	S3	S4	S5	S6	S7	S8
**CFBE41o- cells**	1.7±0.1	36.6±4.7	31.6±5.3	1.1±0.2	1.7±0.3	13.7±1.8	37.0±7.0	34.9±5.0	26.8±5.5	11.7±3.9	0.7±0.1	0.4±0.2
**16HBE14o- cells**	1.3±0.1	43.2±9.1	33.3±7.3	1.4±0.7	1.6±0.7	15.9±4.9	32.5±5.7	34.1±9.4	32.6±3.5	16.5±1.6	1.3±0.2	1.1±0.2

RTN suspensions were nebulised through the NGI. Pre-nebulisation samples, the leftover in the nebuliser chamber and the aerosolised samples retrieved from the different NGI stages were used to assess the percentage of cells transfected by flow cytometry. Ctrl = control; PN = suspension prior nebulisation; LO = leftover in the nebuliser reservoir after completion of the nebulisation; Thr = throat; S1–S8 = stages 1–8 of the NGI, S8 = micro-orifice collector. The results of two independent experiments are expressed as mean ± standard error of the mean. For each experiment, duplicate wells were pooled together.

### Gene expression from nebulised delivery to mice

The reporter gene for β-galactosidase regulated by an EF1α promoter was selected for reporter gene studies *in vivo* to minimize the immune response to hypomethylated CpG repeats found in most plasmid DNA. In addition, the EF1α promoter was shown previously to confer more persistent expression *in vivo* [24], [25].

**Figure 5:**
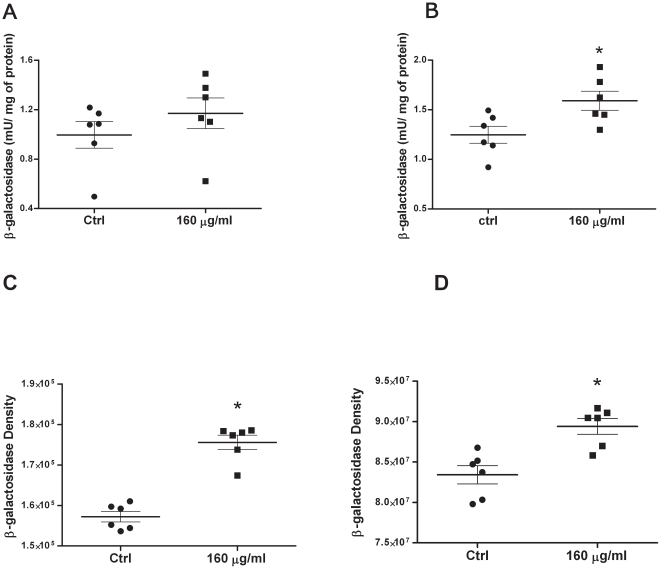
*In vivo* transfection efficiency after a single dose nebulisation in CD1 mice. RTN suspension (6 ml), at a concentration of 160 µg/ml of DNA, was nebulised using the AeroEclipse II BAN in a plexiglass box containing 6 CD1 mice. After 48 h following the nebulisation, the animals were culled and the lungs and the tracheas removed. In the harvested tissues, the levels of β-galactosidase were measured by quantifying the enzymatic activity and by immunodetecting the protein. Control animals were nebulised with the same clinical grade H_2_O in which nanocomplexes were made. **A and B Determination of β-galactosidase enzymatic activity:** The β-galactosidase activity was measured by CPRG assay in both whole lung lysates (A) and in those from the tracheas (B). The activity of enzyme (mU) in the lysates was calculated against the β-galactosidase activity of *Escherichia coli* and normalised for the total protein content. (*) in panel B (tracheas) indicates statistically significant difference (Mann-Whitney test) at 0.05 level (p<0.05). No difference was found in panel A, although there the protein activity showed a similar trend. **C and D Detection of β-galactosidase protein:** The lysates from the whole lungs (C) and the trachea (D) from each mouse were spotted onto PVDF membranes. Dot blots were first probed with goat polyclonal anti-β-galactosidase antibody and then with horseradish peroxidase-labelled rabbit polyclonal anti-goat immunoglobulin. The quantification of duplicate dots per each mouse is shown. The difference between the control group (nebulised with water) and the cohort which received RTNs formed using pCpG-free *lacZ* reporter gene, was statistically significant at the Mann-Whitney test (*, p<0.05).

Groups of six CD1 mice were subjected to whole body nebulisation with RTN suspensions at 160 µg/ml pCpG-free *lacZ* DNA. Mice were killed after 48 h then lung and trachea samples were analysed for β-galactosidase expression by the CPRG assay (Figures 5A and B, respectively), or by β-galactosidase immunodetection on dot blots (Figures 5C and D) in tissue lysates.

The enzymatic activity measured in the lungs (Figure 5A) was not statistically different between the treated (nebulised with RTNs) and the control group (nebulised with water). However, in the tracheas (Figure 5B) the difference between the control and the cohort nebulised with the reporter gene was statistically significant (*p*<0.05). In all the experiments high levels of endogenous β-galactosidase activity was found in the controls, consistent with previous reports [26], [27], [28]. In the lungs, the quantification of the β-galactosidase protein (Figure 5C) showed a more substantial difference than the enzymatic activity. The results in the tracheas appeared consistent between the two different assays (Figures 5B and D).

## Discussion

Despite the many therapies in the pipeline targeting specific defects in *CFTR* transcription and expression or modulation of the channel functions [8], there is still a clinical need for gene therapy-based treatment for cystic fibrosis [29]. In the current study, we aimed to optimise the previously developed molecular conjugates for *in vivo* delivery via aerosolisation. We have described previously the RTN formulation, a modular, self-assembling receptor-targeted nanocomplex (RTN) formulation comprising a mixture of cationic liposomes, a receptor-targeting/DNA-binding peptide and plasmid DNA (D). This formulation displayed receptor-targeted transfection mediated by the peptide, with endosomal release of DNA to the cytoplasm enhanced by the liposome component [30]. In developing the RTN formulation for gene therapy of cystic fibrosis we have demonstrated its capacity to transfect non-dividing epithelial cells in vitro [31], and optimised the lipid and peptide components of the formulation for gene delivery to airway epithelial cells [16], [32]. *In vivo* studies performed in rats [33], mice [15], [34] and pigs [35] demonstrated high efficiency of transfection in airway epithelium combined with low inflammatory potential.

The RTN formulation used in this study is the same as that delivered to mice in recent studies by intratracheal instillation [15]. The peptide contains the targeting motif SERSMNF, an epithelial targeting peptide identified by our group in phage display biopanning experiments [16]. SERSMNF is almost identical to the ICAM-1 targeting sequence of rhinovirus a respiratory pathogen. The K_16_ region of the peptide is important for DNA packaging. The lipid component is DHDTMA/DOPE where DHDTMA is a cationic lipid based on a glycerol backbone with two unsaturated C16 alkyl chains linked by diether linkages [32]. RTN particles target cells by both ICAM-1 receptor and by non-specific cationic properties, and are internalised endocytically. The lipid component mediates endosomal release of the DNA, which is subsequently transported to the nucleus. This step may be facilitated by the K_16_ peptide domain interacting with nuclear importins. Thus there is a high degree of synergy between the lipid and peptide components that contribute to its transfection efficiency.

Aerosol therapy, with mucolytics, antibiotics and other treatments, is widely used to treat CF patients and would be the best option for gene therapy interventions. Different nebulisers were compared to determine if their mode of operation resulted in delivery of RTNs likely to reach the lower airways whilst retaining their transfection efficiency.

Three nebulisers were selected for comparison. The Aeroneb® Pro was tested as it has been shown to generate aerosol particles appropriate for delivery to the lower respiratory tract and it has the added advantage of yield efficiency, due to the vibrating mesh design. Unfortunately, in these experiments, RTN delivery efficiency of the Aeroneb® Pro was significantly less than that of the two jet nebulisers. The pores in the mesh appeared to block during nebulisation. The reason for this is unclear, but may be related to the high positive charge of the RTNs adhering to the mesh. The PARI-LC Plus is a commonly used breath-enhanced nebuliser that has an advantage over conventional jet nebulisers in that the amount of suspension delivered during inspiration is greater. This is achieved through a valve on the top of the nebuliser opening due to the negative pressure developed by the patient during inspiration pulling more air through the nebuliser and with it more aerosol particles. On expiration, the valve closes and during this time the nanocomplex suspension is lost from the device in a similar amount to that of a standard jet nebuliser. The AeroEclipse II BAN is a newly designed nebuliser that only nebulises during inspiration when the negative pressure pulls a central ‘column’ down that enhances the Bernoulli effect of the rapidly expanding gas from the compressor. This results in fluid being pulled up from the reservoir for nebulisation only during inspiration. Unlike the PARI-LC Plus no nanocomplex suspension is lost during expiration from the AeroEclipse resulting in more RTNs being available for inhalation. The option of operation in breath-actuated mode would be extremely useful in the clinical setting as the aerosol is produced in response to the patient inspiration rate.

As both nebulisers otherwise produced aerosolised nanocomplexes that retained their transfection properties the AeroEclipse II BAN was chosen for the remaining experiments. The AeroEclipse nebuliser was examined in more detail for DNA degradation or particle disruption during the aerosolisation process, for biophysical properties, for the ability of transfecting a sufficient number of cells to be clinically relevant and for its efficacy in a small animal model.

The nebulised nanocomplexes not only retained the ability to transfect, but the integrity of their DNA content was also preserved (Figure 2) in agreement with what was previously reported by others [36], [37]. Those RTNs showed enhanced colloidal stability (Figure 3), therefore were less prone to aggregation or flocculation.

Several reports indicate that as little as 6–25% of epithelial cells with restored *CFTR* functions are sufficient to correct the disease [38], [39], [40]. The result in figure 4 not only quantified the number of cells transfected by nanocomplexes, but provided also a good indication at which level of the airways the gene expression could be expected. The NGI data predicted that the majority of cells that are likely to receive the reporter gene resided in the central (trachea) and lower ciliated thoracic portion of the respiratory tree, and not in the alveoli or in the upper airways.

The AeroEclipse aerosolised RTNs demonstrated productive gene expression in most of the C57BL6 mice lungs and luciferase expression was found in ciliated cells in immunohistochemical sections of the trachea. *In vivo* experiments in another murine strain, CD1 mice, confirmed that the delivery of the gene occurred both in the trachea and in the lungs and demonstrated that the delivery and the expression are not strain related. The CD1 mice are a bigger strain than the C57BL6, and were able to reach easily the nozzle, thus were more effective at breathing in the aerosol. No changes were observed in the breathing pattern of either strain during nebulisation. Apoptotic activity of the RTNs on the epithelium as well as the inflammation associated to pCILuc was assessed in a previous study [15]. It has been previously reported that plasmids devoid of unmethylathed CG dinucleotides (CpG) were less inflammatory [41].

We used pCpG-free *lacZ* plasmid-based RTNs to nebulise CD1 mice and the expression of the reporter gene was measured with different methods in both lungs and tracheas. Although similar trend was shown in both tissues, the enzymatic activity in the lungs of treated animals was not statistically different when compared to the controls. The interpretation of the data was complicated because β-galactosidase activity is more difficult to detect in lung tissue than in other tissue types, especially with low gene expression levels [42]. Although the *lacZ* reporter gene delivered is derived from *Escherichia coli*, mammalian tissues also express endogenous β-galactosidase or enzymes with similar catalytic (metabolic) activity, which determines high background level [28], [43], [44]. A further hurdle is due to the presence of blood in tissues, which interferes with the read-out of several colorimetric substrates as the haemoglobin has a broad spectrum of absorption [44]. To minimise the potential misinterpretation of the results (Figure 5) when measuring the enzymatic activity, we used chloro-phenol-red-β-D-galactopyranoside (CPRG) as substrate, which is more sensitive and accurate [43], and to detect the protein (dot blots) we probed the tissue lysates with an antibody specific for the bacterial β-galactosidase.

In this study we have demonstrated that RTN formulations in water can be nebulised with optimal efficiency levels by AeroEclipse II BAN jet nebuliser. The nebulised aerosol containing the nanocomplexes was collected in the cups of a NGI and showed to have an aerodynamic size in the range of approximately 2–8 µm. Aerosol particles of this size would be compatible with deposition in the upper part of the lower respiratory tract rather than deeper in the lung, which correlates with the region of highest *CFTR* expression in the lung and so is the required target site for delivery of CF gene therapy [45]. Nebulised nanocomplexes retained their biophysical properties, and pDNA extracted from the nanocomplexes was present as intact circular molecules. *In vitro* transfection experiments demonstrated that the nebulised nanocomplexes and pDNA retained their functionality for gene expression in transfected cultures of bronchial epithelial cells. *In vivo* delivery of RTNs by whole body nebulisation with the AeroEclipse II BAN nebuliser, led to the successful transfection of murine lungs, which was particularly evident in ciliated cells of the upper airways. Further studies are needed to assess the delivery of the corrective gene *in vivo*, and efficacy after repeated dosing.

Our findings may foster new opportunities for the therapeutic intervention of cystic fibrosis. The dynamic airway plasticity requires repeated applications and the receptor-targeted nanocomplexes presented in the current work may offer an advantage for clinical translation.

## Materials and Methods

### RTN formulation

Receptor-targeted nanocomplexes (RTN) were prepared as described elsewhere [15]. Briefly, liposomes formulated with DHDTMA/DOPE were mixed with the peptide E (K_16_GACSERSMNFCG) and plasmid DNA, all dissolved in water (Baxter S.A., Lessines, Belgium), at the weight ratio of 1.5∶4∶1 (L∶P∶D). Complexes were then left to assemble for at least 1 h at room temperature before being used either for transfections or for nebulisation experiments.

DHDTMA chloride [1-propanaminium, *N,N,N*-trimethyl-2,3-bis(11*Z*)-hexadecenyloxy)-chloride) [46] and DOPE (1,2-dioleoyl-sn-glycero-3-phosphoethanolamine), liposomes were prepared at 1∶1 molar ratio as described previously [32], or purchased from Avanti Polar Lipids Inc. (Alabaster, AL, USA). Peptide E was synthesised by Zinsser Analytic (Maidenhead, UK). Plasmid DNA encoding for enhanced green fluorescent protein (pEGFP-N1) was from Clontech (Clontech-Takara Bio Europe, Saint-Germain-en-Laye, France), whereas plasmid pCILuc consists of pCI (Promega, Southampton, UK) carrying a luciferase gene driven by the CMV promoter-enhancer [16]. The pCpG-free *lacZ* plasmid (Invivogen, San Diego, CA, USA) encodes for β-galactosidase under the EF1α promoter, but is devoid of CpG dinucleotides.

### Cell transfections

The normal bronchial (16HBE14o-) and the CF (CFBE41o-) cell-line, kindly provided by Dieter Gruenert (California Pacific Medical Center Research Institute, San Francisco, CA, USA), were maintained in Minimum Essential Medium Eagle's modification (Sigma, Poole, UK) at 37°C in a humidified atmosphere with 5% CO_2_. Tissue culture medium was supplemented with 10% heat-inactivated foetal bovine serum (FBS, Invitrogen, Paisley, UK), 2 mM L-glutamine (Invitrogen) and 0.1 mM non-essential amino acids (Sigma).

*LacZ* expression was measured in cell extracts with a β-glo assay (Promega Corporation Madison, WI, USA) using a FLUOstar Optima luminometer (BMG Labtech, Aylesbury, UK), according to the instructions of the manufacturer. The results were standardised for protein content using a Bradford protein assay (Bio-Rad Laboratories, Hercules, CA, USA). Luminescence was expressed as relative light units (RLU) per milligram of protein.

### Nebulisations and Next Pharmaceutical Generation Impactor (NGI)

The nebuliser AeroEclipse II BAN was a kind gift of Trudell Medical International Europe Ltd (Manchester, UK), PARI-LC Plus was purchased from PARI Medical Ltd (West Byfleet, UK), while Aeroneb® Pro was kindly provided by Aerogen Ltd (Galway, Ireland). For the nebulisation studies, 3 ml of nanocomplexes (unless stated otherwise), prepared in H_2_O, were added to the sample chamber of the device. The nebuliser was then connected to the compressor and to the throat of the NGI (Copley Scientific Ltd, Nottingham, UK). The nebulisation was performed for 10 min. The NGI was operated at a flow rate of 15 L/min, after cooling at 4°C for at least 1 h. The AeroEclipse was set on continuous operational mode, thus providing a near constant delivery rate independent from respiratory activity. After nebulisation, the residual amount of nanocomplex suspension was measured and collected. All the NGI stages and the throat were carefully rinsed with 1 ml of clinical grade H_2_O (Baxter). Aliquots of the recovered material were used for cell transfections, particle sizing and for DNA quantification experiments.

### Flow cytometry

RTN formulations containing the pEGFP-N1 plasmid (Clontech-Takara Bio Europe) were nebulised through the NGI. Pre-nebulisation samples (PN), the leftover (LO) in the nebuliser reservoir upon completion of the nebulisation and the aerosolised samples, collected from the throat and the different stages of the NGI and diluted with 1 ml of clinical grade H_2_O, were used for transfections. 30 µl of each sample was added to 500 µl OptiMEM (Invitrogen) and then to cells seeded in 24-well plates, grown at 80% confluence. After 48 h incubation, the cells were trypsinised and filtered with a cell strainer (Ø 70 µm). Cells of duplicate wells were pooled together in PBS containing 3% FBS, before analysing EGFP expression by flow cytometry (LSRII, DB Bioscience Europe, Oxford, UK). Cell viability was determined by propidium iodide staining (Sigma) and at least 10,000 live cells were acquired.

### Quantification of DNA released on agarose gel

RTN formulations were nebulised as described above. All the samples pre- and post-nebulisation, and from the different NGI stages were collected. The DNA was released from the nanocomplexes as described previously [47]. Briefly, 100 µl of samples were mixed with the same volume of 2× DNA release buffer (20 mM Tris-Cl, pH 8.0, 200 mM NaCl, 50 mM EDTA and 1% SDS) and incubated at 50°C for 35 min. The DNA was then purified with the Wizard® DNA Clean-Up System (Promega) according to the instructions of the manufacturer. 25 µl of each sample was loaded and run onto 1% agarose gel. The gels were visualised under UV light and the images were used for DNA quantification. The band densities, following the rolling ball background subtraction set at 20, were analysed on ImageJ programme (http://www.NIH.gov) and the yield of the recovered DNA was calculated.

The yield was expressed as percentage of the sum of the density of DNA (ρ) in each lane corrected for the volume of the sample collected (V), divided by the density of DNA of the starting nanocomplex suspension (ρ_PreNeb_) also corrected by the volume (V_PreNeb_).
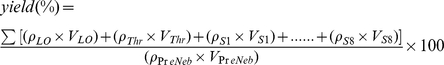



### Size and ζ potential of the RTN complexes

RTN complexes, freshly prepared by using 160 µg/ml of either pCILuc or pCpG-free *lacZ* plasmid, or the samples collected from the NGI cups were diluted 100-fold in DNAse- and RNAse-free water (Invitrogen) to a final volume of 500 µl.

Nanocomplex sizes and ζ potentials were determined by dynamic light scattering and by laser Doppler anemometry, respectively, using a Nano ZS Zetasizer (Malvern Instruments, Malvern, UK) with the following specifications: automatic sampling time of 10 measurements/sample, refractive index of 1.330, dielectric constant 78.5, viscosity 0.8872 cP and temperature of 25°C. ζ potential settings were calibrated against the standard (−68 mV±6.8 mV). A total of three measurements per each sample were automatically performed.

### *In vivo* nebulisation

All animals were handled in strict accordance with good animal practice as defined by the UK Home Office Animal Welfare Legislation, and all animal work was approved by the Institutional Research Ethics Committee (Institute of Child Health, University College London, UK) and performed under Home Office project license number 70/7073.

To determine the effectiveness of the different nebulisers, 2 cohorts of 9 female mice of C57BL6 strain (Charles River, UK), were restrained in the Plexiglas box, with 2 apertures, one of which was connected to the nebuliser, while in the second outlet a filter was installed. Once acclimatised, the mice were nebulised with 2 ml of RTN suspension, containing 160 µg/ml pCILuc DNA, over maximum 30 min. The aim of this procedure was to allow comparison of *in vivo* delivery with the AeroEclipse II BAN and Aeroneb® Pro. All the nebulisers produced an aerosol mist, which filled the air within the cage, though the Aeroneb® Pro blocked frequently. Mice were sacrificed at 24 h, the lungs were perfused, resected and 7 were stored at −80°C, while 2 were fixed for immunohistochemistry. Tissues were defrosted in ice, submerged in reporter gene assay lysis buffer (RLB, Roche, Basel, Switzerland), homogenised with an IKA homogeniser (IKA, Staufen, Germany) and centrifuged at 18,890× *g* for 10 min at 4°C. The supernatants were removed and centrifuged at 18,890× *g* for a further 10 min at 4°C before measuring the luciferase activity.

To assess the vector delivery using pCpG-free *lacZ* reporter gene, CD-1 female mice of 6–8 weeks of age (Charles River, UK), weighing 28–30 g, were confined in the same Plexiglas box mentioned above. The AeroEclipse nebuliser was filled with 6 ml of RTN suspension and set on continuous operational mode. All animals were exposed to the aerosol mist containing pCpG-free *lacZ* DNA at a concentration of 160 µg/ml, or H_2_O as control, for about 30 min, respectively. 48 h after nebulisation, all the 6 animals of each cohort were sacrificed. The lungs and tracheas were collected and stored at −80°C. Tissues were then defrosted on ice, added into RLB (Roche), subjected to hard tissue homogenisation with Precellys 24 (Bertin Technologies, France) and centrifuged at 18,890× *g* for 3 min at 4°C. The supernatant was collected and centrifuged at 18,890× *g* for 10 min at 4°C. The samples were then stored at −20°C before biochemical analysis for the expression of β-galactosidase.

### Luciferase activity detection

Luciferase activity in the tissue lysates was measured over 30 s using the Luciferase Assay System (Promega) in a FLUOstar Optima luminometer (BMG Labtech). The amount of protein present in each transfection lysate was determined with the Bio-Rad protein assay reagent (Bio-Rad Laboratories). Luciferase activity was expressed as relative light units (RLU) per milligram of protein. Two million RLU/30 s corresponds to 1 ng of luciferase.

Two of C57BL6 mice nebulised with 2 ml of RTN formulations containing pCILuc were used for luciferase immunohistochemistry. After killing, the lungs and tracheas were fixed in 4% paraformaldehyde. Sections were analysed for firefly luciferase expression, 5 µm thick sections were dewaxed, rehydrated, and blocked with hydrogen peroxide and normal rabbit serum and then processed as described previously [34].

### Biochemical measurement of β-galactosidase activity

The β-galactosidase enzymatic activity was assessed by CPRG (chloro-phenol-red-β-D-galactopyranoside) assay [48], [49]. 50 µl of tissue lysate was added to 150 µl of 1 mg/ml CPRG solution (60 mM NaH_2_PO_4_, 1 mM MgSO_4_, 10 mM KCl, 50 mM β-mercaptoethanol). The β-galactosidase standard curve was prepared with a 2-fold dilution of β-galactosidase enzyme (Sigma) in 100 mM phosphate buffer, pH 7.3, 10 mM MgCl_2_, and 10 mM β-mercaptoethanol, starting from 100 mU. The 96-well plates were incubated at 37°C to allow the development of the colour, before stopping the reaction with 50 µl of 1 M NaCO_3_. The absorbance was read at 595 nm. The enzymatic activity was normalised to total protein concentration as described above.

For the dot blot assay, 5 µl of samples (in duplicate) were added to PVDF membrane (Ø45 µm, Immunobilon P, Millipore UK Ltd. Watford, UK) and a further 25 µl of RLB (Roche) was added. The vacuum was kept on for 1 h until the dots were completely dried. The membrane was left overnight in PBS-1% BSA, washed twice with PBS containing 0.05% Tween 20 (Sigma; PBS-Tw), then incubated for 90 min with goat polyclonal anti-β-galactosidase antibody (10 µg/ml in PBS-Tw, AbD Serotec, Kidlington, UK). After 3 washes with PBS-Tw, the dot blot membrane was incubated overnight at 4°C with horseradish peroxidase-labelled rabbit polyclonal anti-goat immunoglobulin (50 ng/ml in PBS-Tw; Dako UK Ltd., Ely, UK). Following further washing, the membrane was developed with ECL (Pierce, Thermo Fisher Scientific - Rockford, IL, USA). Following the rolling ball background subtraction set at 50, the densities of the dots were analysed on ImageJ programme (http://www.NIH.gov).

### Statistics

Data were analysed using GraphPad 5.0 InStat (GraphPad Software, Inc., La Jolla, CA) and are expressed as mean ± SEM, unless otherwise indicated. Population medians were interpreted using a non-parametric (Mann-Whitney) test. Differences were considered to be statistically significant for at least p<0.05.
